# Baicalin, a Potent Inhibitor of NF-κB Signaling Pathway, Enhances Chemosensitivity of Breast Cancer Cells to Docetaxel and Inhibits Tumor Growth and Metastasis Both *In Vitro* and *In Vivo*

**DOI:** 10.3389/fphar.2020.00879

**Published:** 2020-06-17

**Authors:** Anqi Zeng, Xin Liang, Shaomi Zhu, Chi Liu, Xiaohong Luo, Qinxiu Zhang, Linjiang Song

**Affiliations:** ^1^School of Medical and Life Sciences/Reproductive & Women-children Hospital, Chengdu University of Traditional Chinese Medicine, Chengdu, China; ^2^Department of Otolaryngology, Hospital of Chengdu University of Traditional Chinese Medicine, Chengdu University of Traditional Chinese Medicine, Chengdu, China; ^3^Institute of Translational Pharmacology and Clinical Application of Sichuan Academy of Chinese Medical Science, Chengdu, China

**Keywords:** baicalin, metastasis, chemosensitivity, NF-κB, breast cancer

## Abstract

**Objective:**

The aim of this study is to investigate the anti-cancer activity and sensibilization of baicalin (BA) against breast cancer (BC) cells.

**Methods:**

The anti-proliferation of BA in BC cell lines was evaluated by MTT and colony formation assays. Apoptotic induction of BA was measured by flow cytometry. Wound-healing and transwell assays were exploited to assess migrated and invasive inhibition of BA. Western-blot and immunofluorescence were used to study mechanisms of anti-migration and sensibilization of BA. Anti-tumor and anti-metastasis effects of BA were evaluated in subcutaneous and pulmonary metastasis mouse model of BC cells.

**Results:**

BA significantly suppressed proliferation and induced apoptosis of BC cells in a concentration- and time-dependent manner. Additionally, BA induced cell apoptosis *via* the mitochondria-mediated pathway, as evidenced by cellular induction of reactive oxygen species and upregulated expression of the Bax/Bcl-2 ratio. The overall expression and nuclear translocation of NF-κB signaling pathway in BC cells were dramatically inhibited by treatment with BA. BA significantly suppressed abilities of migration and invasion in BC cells. Notably, BA sensitized BC cells to docetaxel (DXL) by suppressing the expression of survivin/Bcl-2. BA also retarded tumor growth and triggered apoptosis of tumor cells in a tumor mouse model of 4T1 cells. Furthermore, pulmonary metastasis of BC cells was distinctly suppressed by BA in a tumor mouse model of 4T1 cells.

**Conclusion:**

BA effectively triggered apoptosis, inhibited metastasis, and enhanced chemosensitivity of BC, implying that BA might serve as a promising agent for the treatment of BC.

## Introduction

Breast cancer (BC) is the second leading cause of cancer-related mortality among women (DeSantis et al., 2019), accounting for 208,8849 new cases and 626,679 deaths worldwide in 2018 (Ferlay et al., 2019). Meanwhile, it is estimated that there will be 268,600 new cases of invasive BC and 41,760 deaths among American women in 2019 (Siegel et al., 2019). Even though great advances have been achieved in the treatment of BC over the past few decades, the overall survival of BC patients remains unsatisfactory (Wimmer et al., 2019). BC, especially triple-negative BC, which is defined by no or low expression of estrogen receptor, progesterone receptor, and human epidermal growth factor receptor 2, usually metastasizes from primary tumors to distant sites, such as the brain, lung, liver, and bone, thereby resulting in a poor prognosis and high mortality (Cheung and Ewald, 2014; Couch et al., 2015; Boire et al., 2020). Multi-drug resistance, which is also referred to as low chemosensitivity, is regarded as a notable obstacle to the treatment of BC (De Angelis et al., 2019). Therefore, novel agents with improved therapeutic effects against BC are urgently needed.

Nuclear factor kappa B (NF-κB), which was identified as a DNA-binding protein in 1986, has been widely implicated in many human diseases, including inflammatory disorders, viral infections, metabolic diseases, cell proliferation, and oxidative stress, among others (Sen and Baltimore, 1986; Baldwin, 1996; Kumar et al., 2004; Wong and Tergaonkar, 2009). Increasing evidence has demonstrated that constitutive activation of NF-κB promotes the progression of human cancers of the breast, colon, rectum, stomach, and lung (Karin and Lin, 2002; Van, 2007). Numerous genes involved in tumor cell apoptosis (B-cell lymphoma 2 [Bcl-2], inhibitor of apoptosis proteins, caspase-3) (Li and Sethi, 2010; Gyrd-Hansen and Meier, 2010), cycling (cyclin D1) (Cao et al., 2007), proliferation (cyclooxygenase-2) (Poligone and Baldwin, 2001), angiogenesis (vascular endothelial growth factor, interleukin 8) (Huang et al., 2001), and epithelial-mesenchymal transition (EMT)-related metastasis (López-Novoa and Nieto, 2009) are influenced by constitutive activation of the NF-κB signaling pathway. Paradoxically, clinical studies have revealed that chemotherapeutic agents, such as docetaxel (DXL), not only induce apoptosis of BC cells, but also activate the NF-κB signaling pathway, which suppresses activation of the caspase cascade by upregulating the expression levels of anti-apoptotic proteins, including survivin and Bcl-2 (Chu et al., 1997; Baldwin, 2001). Abnormal activation of NF-κB by chemotherapeutic drugs leads to low chemosensitivity or even drug resistance of cancer cells. Various studies have found that inhibition of the NF-κB signaling pathway can induce apoptosis, suppress proliferation and invasion, and increase chemosensitivity of many types of cancer cells (Kani et al., 2013; Shostak and Chariot, 2015; Capece et al., 2020). Thus, NF-κB could serve as a promising therapeutic target for the treatment of BC.

**Figure 1:**
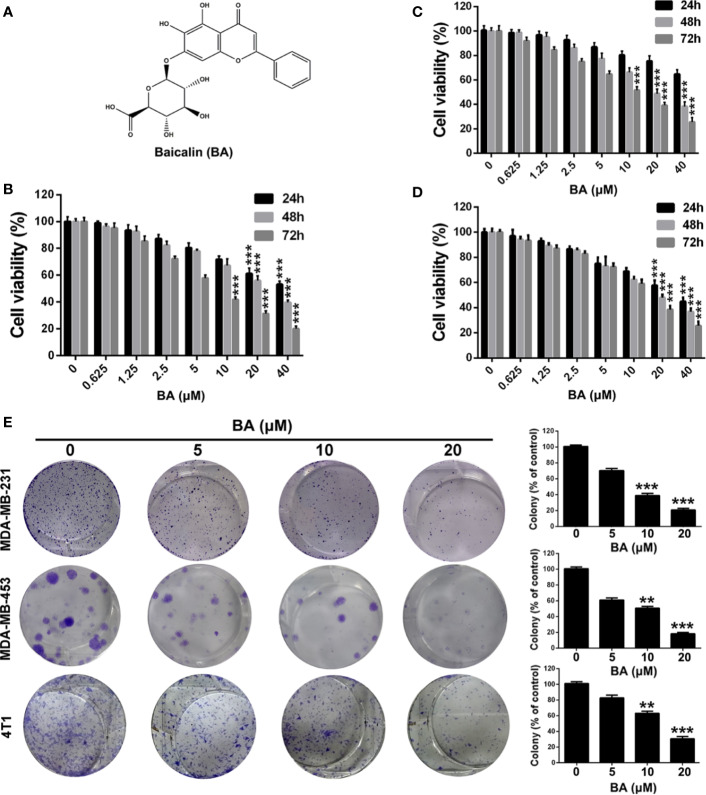
BA exhibited cytotoxicity on breast cancer cells. **(A)** Chemical structure of BA. **(B–D)** Viabilities of MDA-MB-231, MDA-MB-453, and 4T1 cells were measured by MTT assay after treatment with different concentrations (0, 0.625, 1.25, 2.5, 5, 10, 20 and 40 μM) of BA for 24, 48, and 72 h. **(E)** Proliferation of MDA-MB-231, MDA-MB-453, and 4T1 cells were evaluated by colony formation assay after treatment with different concentrations (0, 5, 10, and 20 μM) of BA. Significant differences are indicated as follows: **P < 0.01; ***P < 0.001.

Baicalin (BA, **Figure 1A**), a flavone compound extracted from the dry root of the herb *Scutellaria baicalensis*, exhibits many biological properties, including antibacterial, antioxidant, anticarcinogenic, and anticancer activities (Li-Weber, 2009; Chen et al., 2014; de Oliveira et al., 2015; Fang et al., 2020). BA has been reported to be non-toxic in animals and safe for use in humans (Avila-Carrasco et al., 2019). Accumulating evidence have demonstrated that BA triggers apoptosis and inhibits the proliferation, migration, and invasion of cancer cells of the lung, colon, and breast (Gong et al., 2017; Avila-Carrasco et al., 2019). Jin et al. demonstrated that BA alleviated benign prostate hyperplasia through androgen-dependent apoptosis (Jin and An, 2020), while Chung et al. revealed that BA inhibited NF-κB-mediated EMT of human breast epithelial cells (Chung et al., 2015). Additional studies have found that BA attenuated lipopolysaccharide-induced inflammation, protects against ethanol-induced chronic gastritis, and ameliorated myocardial ischemia-reperfusion injury *via* suppression of the NF-κB signaling pathway (Ji et al., 2019; Luan et al., 2019; Wu et al., 2019). In view of the key role of NF-κB in BC, we hypothesized that BA, which is an effective inhibitor of the NF-κB signaling pathway, could be serve as a promising agent for the clinical treatment of BC.

The results of the present study demonstrated that BA inhibited proliferation and induced mitochondria-mediated apoptosis of BC cells. Meanwhile, the migratory and invasive capabilities of BC cells were significantly inhibited by BA *via* the NF-κB/EMT signaling pathway. Moreover, BA enhanced the chemosensitivity of BC cells to DXL *via* inhibiting activation of the NF-κB signaling pathway and suppressed tumor growth and pulmonary metastasis in a mouse model of BC.

## Materials and Methods

### Materials and Reagents

BA (J&K Scientific, Beijing, China) with purity of >98%, as determined by high-performance liquid chromatography, was dissolved in dimethyl sulfoxide (DMSO) as a stock solution (40 mM) and stored at −20°C for further use. A solution of 0.1% DMSO served as a control. Rhodamine 123 (Rh123), 3-(4,5-dimethylthiazol-2-yl)-2,5-diphenyltetrazolium bromide (MTT), and 2′,7′-dichlorofluorescin diacetate (DCFH-DA) were obtained from Sigma-Aldrich Corporation (St. Louis, MO, USA). An annexin V-fluorescein isothiocyanate apoptosis detection kit was purchased from 4A Biotech Co., Ltd. (Beijing, China). Antibodies against cleaved caspase-3, Bax, Bcl-2, phosphorylated IκBα (p-IκBα), IκBα, NF-κB p65, E-cadherin, N-cadherin, survivin, and β-actin were purchased from Cell Signaling Technology (Beverly, MA, USA). Anti-Ki-67 mouse monoclonal antibody was obtained from EMD Millipore (Billerica, MA, USA).

### Cell Lines

The human BC cell lines MDA-MB-231 and MDA-MB-453, and the murine mammary cancer cell line 4T1 were purchased from the American Type Culture Collection (Manassas, VA, USA). Cells were cultured in Dulbecco’s modified Eagle’s medium or Roswell Park Memorial Institute 1640 medium containing 10% fetal bovine serum (FBS; Caoyuan Lvye Biological Engineering Materials Co., Ltd., Hohhot, China) and 1% antibiotics (penicillin and streptomycin) at 37°C under a humidified atmosphere of 5% CO_2_/95% air.

### Cell Viability and Colony Formation Assay

The MTT assay was used to assess the viability of BC cells. In brief, 2,000–5,000 cells were seeded into the wells of a 96-well culture plate, cultured for 12 h, and then exposed to various concentrations (0–40 μM) of BA for 24, 48, or 72 h. Afterward, 20 μl of MTT solution (5 mg/ml) was added to each well and the cells were incubated at 37°C for an additional 3 h. Subsequently, the supernatant was replaced with 150 μl of DMSO. Finally, the absorbance of each well at 570 nm was measured using a SpectraMax® M5 Multi-Mode Microplate Reader (Molecular Devices, Sunnyvale, CA, USA). Each experiment was conducted at least three times.

For the colony formation assay, 200–600 cells were seeded into the wells of a six-well plate, cultured for 12 h, and then treated with different concentrations (0, 5, 10, or 20 μM) of BA for 12 days. Cell culture medium containing different concentrations of BA was refreshed every 3 days. Finally, the cells were washed with phosphate-buffered saline (PBS), fixed with methanol for 10 min, and stained with crystal violet (0.5%, w/v) for 20 min. Afterward, the cells were photographed and counted under a light microscope (Olympus Corporation, Tokyo, Japan) equipped with a digital camera.

### Cell Apoptosis

BC cells, which were seeded into the wells of a six-well plate, were treated with different concentrations (0–40 μM) of BA for 48 h, then harvested, washed three times with ice-cold PBS, and stained with the use of an Annexin V/propidium iodide (PI) dual labeling apoptosis detection kit (Enzo Life Sciences, Inc., Farmingdale, NY, USA) in accordance with the manufacturer’s guidelines. Finally, apoptotic cells were detected by flow cytometry (FCM) (BD Biosciences, Franklin Lakes, NJ, USA).

### Measurement of Mitochondrial Membrane Potential and Cellular Levels of Reactive Oxygen Species (ROS)

BC cells were seeded into the wells of a six-well plate and cultured for 12 h. After exposure to various concentrations of BA for 48 h, the cells were harvested, washed with PBS, and incubated with 5 μg/ml of Rh123 solution at 37°C for 25 min in the dark. Finally, the mitochondrial membrane potential of the treated cells was measured by FCM.

For detection of cellular ROS levels, following exposure to various concentrations of BA for 48 h, BC cells were harvested, washed with PBS, and incubated with DCFH-DA (10 μM) for 20 min at 37°C in the dark. Finally, ROS levels of the treated cells were measured by FCM.

### Wound-Healing Assay

After culturing in the wells of a six-well plate, BC cells at about 80–90% confluency were scraped with a sterile 10-μl pipette tip. Subsequently, the culture medium was replaced with new medium containing 1% FBS and different concentrations of BA. Images of BC cells were obtained at 0 and 48 h under a light microscope (Olympus Corporation) equipped with a digital camera.

### Transwell Invasion Assay

The transwell invasion assay was used to assess the invasive capability of BC cells. Briefly, MDA-MB-231 (1 × 10^5^) and 4T1 (5 × 10^4^) cells suspended in 100 μl of serum-free medium were added to the upper chamber, which was precoated with 40 μl of Martrigel. Meanwhile, 600 μl of cultured medium containing 10% FBS was added to the lower chamber. Different concentrations of BA were added to the medium both in the upper and lower chambers. After 48 h, the invasive cells located on the lower membrane were fixed with methanol and stained with crystal violet (0.5%, w/v) for 20 min. Photos of the invasive cells were obtained under a light microscope (Olympus, Japan) equipped with a digital camera. Invasive cells from four independent areas were counted in order to calculate the invasion rate.

### Western Blot Assay

BC cells exposed to different BA concentrations were harvested, washed with ice-cold PBS, and lysed with radioimmunoprecipitation assay buffer (Beyotime Institute of Biotechnology, Beijing, China) in order to obtain the total protein content. Equal amounts of protein samples from different treatment groups were separated by sodium dodecyl sulfate-polyacrylamide gel electrophoresis and then transferred onto polyvinylidene diﬂuoride nitrocellulose membranes (EMD Millipore). After incubating with 5% (w/v) non-fat milk at 37°C for 1 h, the membranes containing the target proteins were incubated with the corresponding primary antibodies overnight at 4°C. Afterward, membrane-bound proteins were treated with corresponding horseradish peroxidase-conjugated secondary antibodies and visualized with the use of a chemiluminescence kit (EMD Millipore).

### Immunofluorescence Assay

BC cells cultured on circular glass discs in the wells of 24-well plates were exposed to 20 μM of BA for 24 h and/or 15 ng/ml of TNF-α for 4 h. Then, the cells were fixed with 4% paraformaldehyde for 10 min, permeabilized with 1% Triton X-100 for 15 min, washed three times with ice-cold PBS, and blocked with 5% bovine serum albumin for 1 h. Following overnight incubation with primary antibodies at 4°C, the cells were washed with ice-cold PBS and treated with fluorescein isothiocyanate-conjugated goat anti-rabbit immunoglobulin G secondary antibody (dilution, 1:500; Beyotime Institute of Biotechnology) for 1 h at room temperature. Finally, the cells were stained with 4′,6-diamidino-2-phenylindole (dilution, 1:10,000) for 10 min and photographed with the use of a confocal laser scanning microscope (Leica Microsystems GmbH, Wetzlar, Germany).

### Anti-Tumor Evaluation

All animal experiments in this study were performed according to the National Institutes of Health (Bethesda, MD, USA) guidelines and were approved by the Institutional Animal Care and Treatment Committee of West China Second University Hospital (Animal ethics approval No.: 2018082). Female Balb/c mice (6–8 weeks old) were purchased from Beijing HFK Bioscience Co., Ltd. (Beijing, China). The right flank of each mouse was subcutaneously injected with 1 × 10^6^ 4T1 cells suspended in 100 μl of culture medium containing no FBS or antibiotics in order to establish a xenograft tumor mouse model. At 7 days post-inoculation, once the tumor volume was about 100 mm^3^, the tumor-bearing mice were randomly assigned to one of three treatment groups (control, 25 mg/kg, or 50 mg/kg, n = 3/group). Then, the mice were intraperitoneally injected with different doses of BA every 2 days. Tumor volume (V = 0.5 × LW^2^, where L and W are the tumor length and width, respectively) and body weight were measured and recorded every 2 days. At the end point of the experiment, the mice were euthanized by cervical dislocation. Tumors from the different treatment groups were isolated, photographed, weighed, and fixed with paraformaldehyde for further immunohistochemical analyses. Major organs (heart, liver, spleen, lungs, and kidneys) were also isolated and fixed with paraformaldehyde for histopathological analysis.

### Anti-Pulmonary Metastasis Evaluation

In order to establish a mouse model of pulmonary metastasis, female Balb/c mice were intravenously injected with 5 × 10^5^ 4T1-Luciferase cells suspended in 100 μl of culture medium containing no FBS or antibiotics. Five days after inoculation, the mice were randomly assigned to one of three treatment groups (control, 25 mg/kg, or 50 mg/kg, n = 3/group). Then, the mice were intraperitoneally injected with different doses of BA every 2 days. At 5, 10, and 15 days post-treatment, the mice were anesthetized and intraperitoneally injected with 100 μl of D-luciferin (30 mg/ml in PBS). Then, the bioluminescence of the metastatic lung tumors was analyzed using an IVIS Lumina *in vivo* Imaging System (PerkinElmer, Inc., Waltham, MA, USA). At the end point of the experiment, lungs from each group were isolated, weighed, and the number of metastatic nodules (diameters of >3 and <3 mm) was counted.

### Statistical Analysis

All data are presented as the mean ± standard deviation of three independent experiments. The unpaired two-tailed Student’s *t*-test was used to assess the statistical significance of mean diﬀerences between groups. Combination index was calculated by software of Calcusyn. All statistical analyses were conducted using IBM SPSS Statistics for Windows, version 25.0. (IBM Corporation, Armonk, NY, USA). A probability (*p*) value of <0.05 was considered statistically significant.

## Results

### BA Suppressed Viability and Clonogenicity of BC Cells

To investigate the biological effect of BA, a potent inhibitor of the NF-κB signaling pathway in BC cells, the MTT assay was conducted to evaluate the viability of BA-treated MDA-MB-231, MDA-MB-453, and 4T1 cells. As shown in **Figures 1B–D**, BA significantly inhibited the viability of MDA-MB-231, MDA-MB-453, and 4T1 cells. Furthermore, BA was toxic to BC cells in a time- and concentration-dependent manner. The effect of BA on the colony formation capability of MDA-MB-231, MDA-MB-453, and 4T1 cells was also assessed. As shown in **Figure 1E**, BA treatment distinctly reduced the colony formation capability of BC cells in a concentration-dependent manner. As compared with the control group, the colonies formed by the BA-treatment groups were significantly smaller. These results suggest that BA significantly inhibited the viability and clonogenicity of BC cells in a time- and concentration-dependent manner.

### BA Triggered Mitochondria-Mediated Apoptosis of BC Cells

**Figure 2:**
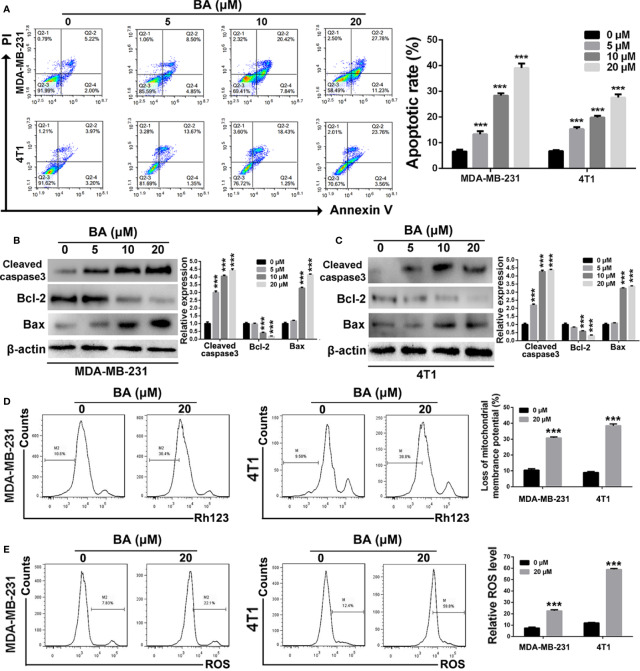
BA triggered mitochondrial-mediated apoptosis in breast cancer. **(A)** Cells apoptosis were detected by Annexin V/PI dual staining assay after treatment with different concentrations (0, 5, 10, and 20 μM) of BA for 48 h. **(B, C)** Changes of apoptotic proteins in MDA-MB-231 and 4T1 cells were detected by western-blot assay. **(D)** Loss of mitochondrial membrane potentials of MDA-MB-231 and 4T1 cells were tested after different treatments. **(E)** The levels of cellular ROS of MDA-MB-231 and 4T1 cells were examined after different treatments. Significant differences are indicated as follows: ***P < 0.001.

The Annexin V/PI dual labeling assay and FCM were performed in order to determine whether BA-induced inhibition of the viability and anti-proliferation capabilities of MDA-MB-231 and 4T1 cells was associated with apoptosis. As shown in **Figure 2A**, the proportion of apoptotic MDA-MB-231 cells increased from 7.22 ± 2.4% to 39.01 ± 2.6% when the concentration of BA was increased from 0 to 20 μM. Meanwhile, as compared with the control group (7.17 ± 1.6%), the proportion of apoptotic 4T1 cells was 27.32 ± 2.3% after treatment with 20 μM BA. To further confirm that BA treatment induced apoptosis of BC cells, western blot analysis was performed to evaluate changes in the expression levels of apoptosis-associated proteins. As shown in **Figures 2B, C**, expression levels of cleaved caspase-3 were significantly upregulated in both MDA-MB-231 and 4T1 cells treated with BA. Furthermore, the expression levels of Bcl-2 in MDA-MB-231 and 4T1 cells were downregulated after treatment with different concentrations of BA, whereas the expression levels of Bax were upregulated, suggesting the induction of mitochondria-mediated cell apoptosis.

To determine whether BA triggers apoptosis of BC cells *via* the mitochondria-mediated pathway, changes to mitochondrial membrane potential and cellular ROS levels were evaluated. As shown in **Figure 2D**, BA dramatically reduced the mitochondrial membrane potential of both MDA-MB-231 and 4T1 cells. Moreover, after treatment with BA, cellular ROS levels in both MDA-MB-231 and 4T1 cells were significantly upregulated as compared to the control group (**Figure 2E**). Taken together, these results demonstrated that BA triggered apoptosis of BC cells in a concentration-dependent manner *via* the mitochondria-mediated pathway.

### BA Impaired the Migratory and Invasive Capabilities of BC Cells *via* the NF-κB Signaling Pathway

**Figure 3:**
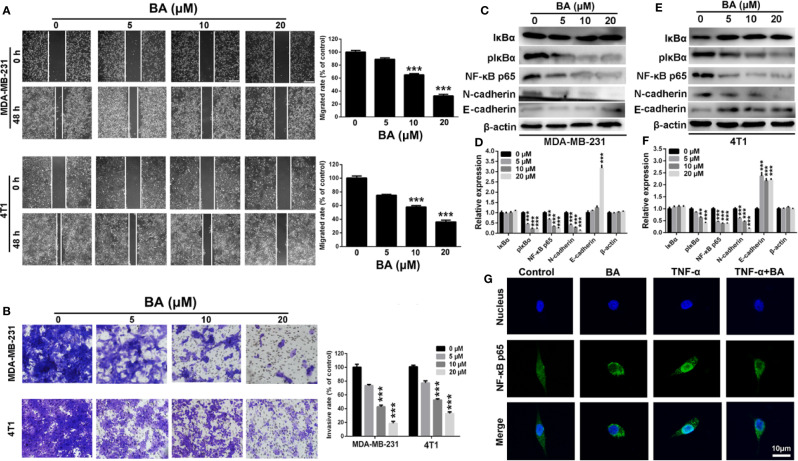
BA inhibited breast cancer migration and invasion *via* NF-κB signaling pathway. **(A)** Migrations of MDA-MB-231 and 4T1 cells were measured by wound-healing assay after treated with different concentrations (0, 5, 10, and 20 μM) of BA for 48 h. **(B)** Invasions of MDA-MB-231 and 4T1 cells were examined by transwell assay after different concentrations (0, 5, 10, and 20 μM) of BA for 48 h. **(C–F)** Changes of NF-κB and EMT related proteins in MDA-MB-231 and 4T1 cells were evaluated by western-blot assay after different concentrations (0, 5, 10, and 20 μM) of BA for 48 h. **(G)** Immunofluorescent analysis of nuclear transportation of NF-κB p65 protein in HCT116 cell. Cells were exposed to 20 μM of BA for 24 h and/or 15 ng/ml of TNF-α for 4 h. Significant differences are indicated as follows: **P < 0.01; ***P < 0.001.

The NF-κB signaling pathway has been implicated in the migratory and invasive capabilities of cancer cells (DiDonato et al., 2012). Therefore, the wound-healing and transwell invasion assays were conducted to assess the anti-migration and anti-invasion effects of BA. As shown in **Figure 3A**, as compared with the control group, the wound-healing capabilities of both MDA-MB-231 and 4T1 cells was strikingly reduced after treatment with BA, indicating the anti-migration effect of BA. As shown in **Figure 3B**, BA treatment distinctly impaired the invasive and migratory capabilities pf MDA-MB-231 and 4T1 cells, as compared to the control cells.

**Figure 4:**
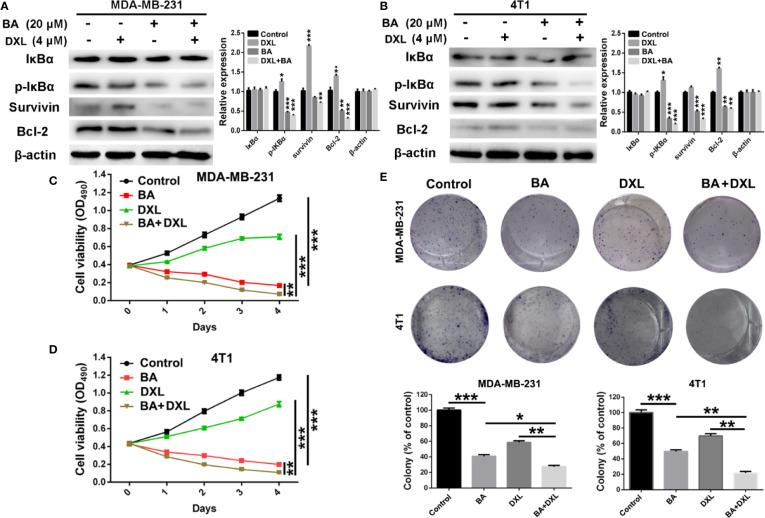
BA sensitized breast cancer cells to docetaxel (DXL) by suppressing NF-κB signaling pathway. **(A, B)** Western-blot analysis of expression levels of apoptotic proteins in MDA-MB-231 and 4T1 cells after treatment with BA (20 μM) and/or DXL (4 μM) for 48 h. **(C, D)** Cell viabilities of MDA-MB-231 and 4T1 were assessed after treated with BA (20 μM) and/or DXL (4 μM) for different times (0, 1, 2, 3, and 4 days). **(E)** Cell proliferations of MDA-MB-231 and 4T1 were evaluated after different treatments. Significant differences are indicated as follows: *P < 0.05; **P < 0.01; ***P < 0.001.

To further investigate the anti-migration and anti-invasion mechanism of BA, western blot analysis was conducted to evaluate changes in the expression levels of metastasis-associated proteins in MDA-MB-231 and 4T1 cells. Compared with the control group, the expression levels of p-IκBα and NF-κB p65, which are essential to the NF-κB signaling pathway, were significantly (*p* < 0.001) upregulated in BA-treated MDA-MB-231 (**Figures 3C, D**) and 4T1 (**Figures 3E, F**) cells, indicating impairment of the NF-κB signaling pathway. Moreover, the expression levels of N-cadherin, which plays a key role in the EMT process, were dramatically downregulated after treatment with BA, as compared with the control group. Meanwhile, the expression level of E-cadherin was significantly upregulated, indicating an inhibition of EMT process of breast cancer cells. Furthermore, the translocation of NF-κB was assessed in BA-treated MDA-MB-231 cells. As shown in **Figure 3E**, NF-κB p65 was expressed in both the cytoplasm and nucleus, resulting from constitutive activation of the NF-κB signaling pathway in MDA-MB-231 cells. When treated with BA, nuclear expression of NF-κB p65 was significantly reduced. More importantly, BA significantly attenuated nuclear translocation of the NF-κB p65 protein, which was induced by treatment with tumor necrosis factor alpha. Collectively, these results confirmed that BA significantly inhibited the migratory and invasive capabilities of BC cells *via* impairment of the NF-κB signaling pathway.

### BA Enhanced the Chemosensitivity of BC Cells to DXL

Various studies revealed that DXL, a first-line drug for the treatment of many cancers, exhibited limited therapeutic efficacy partly due to activation of NF-κB (Kani et al., 2013; Shao et al., 2013; Pan et al., 2016). Therefore, we investigated whether BA, an efficient NF-κB inhibitor, enhanced the chemosensitivity of BC cells to DXL. As shown in **Figures 4A, B**, expression levels of p-IκBα and the anti-apoptotic protein survivin and Bcl-2 were significantly upregulated by treatment with DXL in MDA-MB-231 and 4T1 cells, suggesting that DXL induces chemoresistance in BC cells. However, when treated with BA, DXL-induced activation of NF-κB was weakened, and expression levels of the anti-apoptotic proteins were dramatically downregulated (**Figures 4A, B**). Furthermore, the combination of DXL and BA had antitumor effects against BC cells. As shown in **Figures 4C, D**, DXL combined with BA exhibited more efficient cytotoxicity in MDA-MB-231 and 4T1 cells. In addition, the colony formation capabilities of MDA-MB-231 and 4T1 cells were distinctly inhibited by treatment with both DXL and BA (**Figure 4E**). Importantly, the combination index (CI) of BA and DXL in breast cancer cells were further investigated. As displayed in **Figure S1** and **Table S1**, CI of BA and DXL in MDA-MB-231 cells were 0.90350, 0.53829, and 0.54999, when concentrations of BA increased from 10 to 40 μM and concentration of DXL was kept as 4 μM. The CI in 4T1 cells were 0.94425, 0.57227, and 0.48514, so BA and DXL exhibited a synergetic anti-tumor effect. Collectively, these results demonstrated that BA improved the sensitivity of BC cells to DXL probably *via* suppression of NF-κB activation.

### BA Inhibited BC Growth *In Vivo*

**Figure 5:**
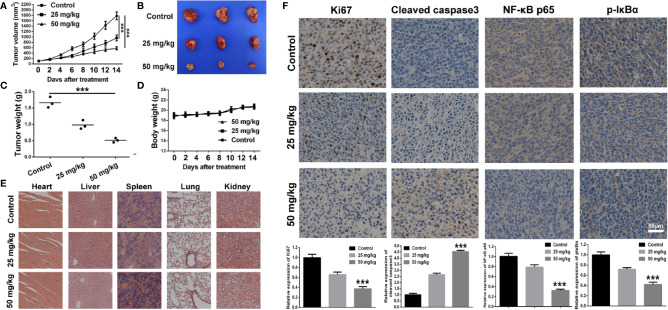
BA suppressed 4T1 tumor growth by inhibiting NF-κB signaling pathway. 1×10^6^ 4T1 cells which was suspended in 100 μl of serum-free cell culture medium were injected into right flank of female Balb/c mouse to establish tumor mouse model. When the tumor volume reached about 100 mm^3^, the tumor-bearing mice were intraperitoneally administrated with vehicle, 25 mg/kg, or 50 mg/kg of BA every 2 days. **(A)** Curve of tumor growth during the process of experiment. **(B)** Tumor images of different treated groups at the termination of animal experiment in the tumor mouse model of 4T1 cells. **(C)** Tumor weight of different treated groups at the termination of animal experiment in the tumor mouse model of 4T1 cells. **(D)** Body weight of mice of different treated groups during the process of experiment. **(E)** Pathological analysis of major organs of subcutaneous tumor-bearing mice after treatment of different doses of baicalin. **(F)** Immunohistochemical analysis of Ki67, Cleaved caspase3, NF-κB p65, and p-IκBα of tumor sections of different treated groups. Significant differences are indicated as follows: ***P < 0.001.

The anti-tumor effect of BA was investigated *in vivo* with the use of xenograft tumor model of 4T1 cells. As shown in **Figure 5A**, as compared to the control group, the tumor growth rate was significantly retarded by treatment with BA at 25 and 50 mg/kg. The individual data for each mouse on tumor growth curve in different treated group were displayed in **Figure S2**, which also demonstrating efficient anti-tumor effect of BA. At the termination of the experiment, tumors from each group were isolated. As shown in **Figures 5B, C**, as compared with the control group, the size and weight of tumors were strikingly reduced after treatment with BA, and there was no significant difference in the body weights of mice among groups (**Figure 5D**). Also, there were no pathological changes to the major organs of mice in the different treatment groups (**Figure 5E**), which demonstrated that BA was not toxic at the organ level.

Immunohistochemical analysis was performed to further explore the anti-tumor mechanism of BA in a 4T1 xenograft tumor model. As shown in **Figure 5F**, the proportion of proliferating (Ki67-positive) cells in the tumor sections was distinctly reduced after treatment with BA. Additionally, as compared with the control group, the expression levels of cleaved caspase-3 in the tumor sections from BA-treated mice were significantly enhanced. Furthermore, the expression levels of NF-κB and p-IκBα, which have been implicated in the NF-κB signaling pathway, were downregulated by treatment with BA. Taken together, BA inhibited BC proliferation and induced apoptosis of tumor cells *via* suppression of the NF-κB signaling pathway.

### BA Suppressed Pulmonary Metastasis of BC

**Figure 6:**
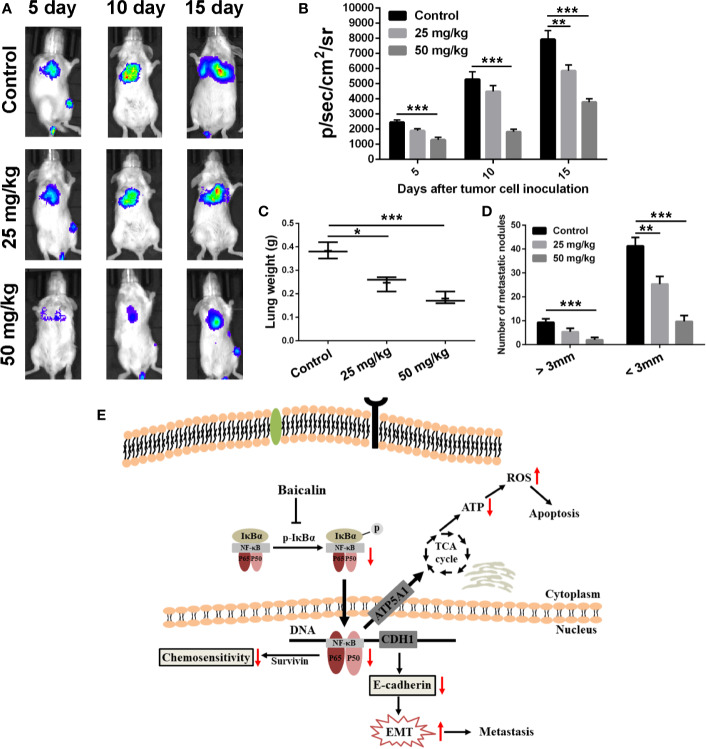
BA inhibited pulmonary metastasis of breast cancer cells. Female Balb/c mice were intravenously injected with 5 × 10^5^ 4T1-Luciferase cells suspended in 100 μl of culture medium containing no FBS or antibiotics to establish pulmonary metastasis tumor mouse model. Five days after inoculation, the mice were intraperitoneally injected with vehicle, 25 mg/kg, or 50 mg/kg of BA every 2 days. **(A)** Bioluminescent images of mice of different treated groups at determined times. **(B)** Statistical analysis of bioluminescent signals of mice of different treated groups at determined times. **(C)** Lungs weight of mice in different treated groups at the termination of animal experiment. **(D)** Numbers of metastatic nodules (>3 mm and <3 mm) in lung tissues were counted at the termination of animal experiment. **(E)** Overview of pathways for baicalin-mediated anti-tumor effects on breast cancer cells. Baicalin induced apoptosis, inhibited metastasis, and enhanced chemosensitivity by NF-κB signaling pathway in breast cancer cells. Significant differences are indicated as follows: *P < 0.05; **P < 0.01; ***P < 0.001.

Inspired by the efficient anti-migration and anti-invasion effects of BA *in vitro*, a pulmonary metastasis tumor model of 4T1 cells was established to evaluate the anti-pulmonary metastasis efficacy of BA *in vivo*. As shown in **Figures 6A, B**, as compared with the control group, the fluorescent intensity, which represents metastatic 4T1-Luciferase cells, in the lungs at 5, 10, and 15 days was significantly reduced after treatment with BA, suggested a pulmonary inhibitory effect of BA in BC. At the end point of treatment, lung tissues of each group were isolated. Strikingly, as compared with the control group, the mean weight of lung tissues was dramatically reduced by treatment with BA (**Figure 6C**). Furthermore, the numbers of metastatic nodules (>3 and <3 mm) of the BA-treatment groups were significantly reduced as compared to that of the control group (**Figure 6D**). Together, these findings demonstrated that BA efficiently inhibited pulmonary metastasis in BC.

## Discussion

BC, which is defined as malignant and highly metastatic, is the most commonly diagnosed cancer and the second leading cause of cancer-related death among women worldwide (DeSantis et al., 2019). In the present study, the anti-tumor activity of BA, a polyphenol compound that is abundant in coffee, was evaluated both *in vitro* and *in vivo*.

First, the MTT and colony formation assays were performed to investigate the cytotoxicity and clonogenicity of BA in BC cells. The study results showed that BA significantly inhibited the viability and colony formation capability of BC cells in a time- and dose-dependent manner. Encouraged by these findings, further experiments were conducted to determine whether BA inhibited BC viability and proliferation through the induction of apoptosis. The Annexin V/PI dual staining results demonstrated that BA distinctly triggered apoptosis of MDA-MB-231 and 4T1 cells. Furthermore, cleaved caspase-3 expression was upregulated by treatment with BA, which confirmed that BA can induce apoptosis of BC cells. Apart from breast cancer cells, BA was reported to suppress the cell cycle progression and proliferation of prostate cancer cells through the CDK6/FOXM1 axis (Yu et al., 2020). Furthermore, Jia et al. demonstrated that BA induced colon cancer cells apoptosis through miR-217/DKK1-mediated inhibition of Wnt signaling pathway (Jia et al., 2019). The intrinsic mechanisms of anti-proliferation and apoptotic induction of BA in breast cancer cells were further investigated.

After treatment with BA, the expression of Bax was upregulated, whereas that of Bcl-2 was downregulated, suggested that BA-induced apoptosis might be associated with the mitochondrial apoptotic pathway, which is particularly relevant to cancer and the typical apoptotic signaling pathway (Liggett and DeGregori, 2017). The results of the present study demonstrated that BA distinctly reduced mitochondrial membrane potential, resulting in destruction of the mitochondrial membrane. Subsequently, the cellular level of ROS, which is abundant in mitochondria, was elevated. The results confirmed that BA triggered apoptosis of BC cells *via* the mitochondria-mediated pathway. Various study demonstrated that ROS production is closely associated with mitochondrial structure (Kim and Song, 2016; Jezek et al., 2018). NF-κB acts as an oxidative stress response transcription factor, and NF-κB pathway can aﬀect mitochondria dynamics (Chen et al., 2016). Vaisitti et al. demonstrated that IT-901, a novel small molecular agent able to suppress the NF-κB subunit c-Rel, exhibited a dose-dependent mitochondrial ROS production and a remarkable decrease of ATP production in chronic lymphocytic leukemia (CLL) cell lines, resulting from decreased expression levels of NF-κB-regulated gene (ATP5A1), involved in tricarboxylic acid cycle or scavenging processes (Vaisitti et al., 2017; Capece et al., 2019). Therefore, icariin might inhibit tricarboxylic acid cycle to promote induction of ROS and then induce mitochondria mediated apoptosis in breast cancer cells by impairing NF-κB pathway.

The NF-κB signaling pathway has been implicated in the migratory and invasive capabilities of cancer cells (Harrington and Annunziata, 2019). Increasing evidence has demonstrated that inhibition of NF-κB can suppress the migration and invasion of various cancer cells (Jiang et al., 2017; Mei et al., 2017; Li et al., 2020). The inhibitory effect of BA on the NF-κB signaling pathway has been widely reported in various diseases (Chen et al., 2014; Duan et al., 2019). Therefore, the aim of the present study was to determine whether BA, a potent inhibitor of the NF-κB signaling pathway, could suppress the migratory and invasive capabilities of BC cells. The results indicated that BA exhibited significant anti-migration and anti-invasion effects in BC cells. Further analysis revealed that the underlying mechanism of BA involved inhibition of the expression of proteins associated with the NF-κB signaling pathway (p-IκBα and NF-κB p65) and EMT (N-cadherin). Furthermore, the translocation of NF-κB p65 was dramatically suppressed by treatment with BA. It is widely reported that BA could alleviate deoxynivalenol-induced intestinal inflammation and oxidative stress damage and protect against ethanol-induced chronic gastritis in rats by inhibiting NF-κB signaling pathway (Ji et al., 2019; Liao et al., 2020). As for tumors, BA, a potent inhibitor of NF-κB, could inhibit migration and invasion in breast cancer by regulating miR-338-3p and MORC4 as well as reversing epithelial-to-mesenchymal transition by targeting β-catenin signaling (Zhou et al., 2017; Duan et al., 2019). Taken together, these findings suggest that BA inhibited the migratory and invasive capabilities of BC cells probably *via* suppression of the NF-κB/EMT signaling pathway.

Resistance to anti-cancer drugs, which results in limited clinical outcomes, is an urgent problem that must be resolved. Many studies have reported that DXL, a first-line drug for the treatment of many types of cancer, exhibited low sensitivity partly due to activation of the NF-κB signaling pathway (Kani et al., 2013; Shao et al., 2013; Pan et al., 2016). In our study, we treated MDA-MB-231 and 4T1 cells with DXL and we observed that the activations of survivin and Bcl-2 were increased and the level of p-IκBα was a little upregulated, which indicated that DXL induced the chemo-resistance of pancreatic cancer cells. So, we hypothesized that inhibition of NF-κB by BA might sensitize cancer cells to DXL. Our findings demonstrated that treatment with BA could downregulate the elevated expression of the anti-apoptotic protein survivin caused by DXL by suppressing the expression of proteins associated with the NF-κB signaling pathway (p-IκBα). In this study, we found that upregulation of p-IκBα by DXL was not so obvious, which probably is because that NF-κB had been excessively activated in MDA-MB-231 and 4T1 cells. More importantly, the combination of BA and DXL exhibited more efficient cytotoxicity and anti-proliferation effects in BC cells. These results indicated that as a potent inhibitor of NF-κB signaling pathway, BA significantly sensitized BC cells to chemotherapy.

Moreover, a xenograft tumor mouse model of 4T1 cells was created to determine whether the anti-tumor efficacy of BA *in vivo* is consistent with the effects *in vitro*. The results showed that BA significantly retarded tumor growth by inhibiting the proliferation and inducing apoptosis of tumor cells. Importantly, BA inhibited the expression of p-IκBα in 4T1 tumor tissues. The expression level of NF-κB p65 was also suppressed by treatment with BA. However, we couldn’t find significant nuclear translocation of NF-κB p65 in tumor section of control group. It is probably that significant nuclear translocation of NF-κB p65 can be obtained only by stimulating with hTNF-α *in vivo*, even though abnormal activation of NF-κB in breast cancer cells. In fact, nuclear translocation of NF-κB p65 could really suppressed by BA, which was demonstrated by immunofluorescence assay. BA significantly attenuated MDA-MB-231 cells nuclear translocation of the NF-κB p65 protein, which was induced by treatment with TNF-α (**Figure 3G**). These results demonstrated that BA inhibited tumor growth by suppressing activation of the NF-κB signaling pathway. The high metastatic potential of BC is associated with its poor prognosis (Insua-Rodríguez and Oskarsson, 2016). In a pulmonary metastasis mouse model of 4T1 cells, BA significantly suppressed metastasis to the lungs. Furthermore, the weight of lung tissues and number of metastatic nodules were both reduced by treatment with BA.

In summary, the results of the present study demonstrated the anti-tumor and anti-pulmonary metastasis effects of BA both *in vitro* and *in vivo* (**Figure 6E**). BA significantly inhibited the viability and proliferation of BC cells by triggering mitochondria-mediated apoptosis. Additionally, BA distinctly suppressed the migratory and invasive capabilities of BC cells by impairing activation of the NF-κB/EMT signaling pathway. More importantly, BA sensitized BC cells to DXL and retarded tumor growth and suppressed pulmonary metastasis. Therefore, BA is a promising candidate to inhibit the growth and metastasis of BC cells.

## Data Availability Statement

The raw data supporting the conclusions of this article will be made available by the authors, without undue reservation.

## Ethics Statement

The animal study was reviewed and approved by the Institutional Animal Care and Treatment Committee of Chengdu University of Traditional Chinese Medicine.

## Author Contributions

QZ and LS designed the research and was responsible for the project conception. LS, AZ, XinL and CL were responsible for statistical analyses and interpretation of the data. LS drafted the manuscript, together with XiaL, SZ and CL. All authors contributed to the article and approved the submitted version.

## Funding

The work was supported by the Chinese Postdoctoral Science Foundation Program (2019M653833XB); Foundation of Science and Technology Department of Sichuan Province (2020YJ0147); Foundation of “apricot grove scholar” of Chengdu University of Traditional Chinese Medicine (2019yky09); Postdoctoral Science Foundation of Chengdu University of Traditional Chinese Medicine (030054080); Foundation of Sichuan Administration of Traditional Chinese Medicine (2018JC010); the Foundation of Health Commission of Sichuan Province (19PJ033); Foundation of Science and Technology Department of Chengdu (2019-YF05-00218-SN).

## Conflict of Interest

The authors declare that the research was conducted in the absence of any commercial or financial relationships that could be construed as a potential conflict of interest.
