# Supplementary material for: Baicalin, a Potent Inhibitor of NF-κB Signaling Pathway, Enhances Chemosensitivity of Breast Cancer Cells to Docetaxel and Inhibits Tumor Growth and Metastasis Both In Vitro and In Vivo
Source: Front Pharmacol. 2020 Jun 17;11:879. doi: 10.3389/fphar.2020.00879 (PMC7311669; doi:10.3389/fphar.2020.00879)
Supplement: Supplementary file 1 [file DataSheet_1.docx]

**Table S1. Combination index of BA and DXL in breast cancer cells**

| **Cell line** | **Dose of BA (μM)** | **Dose of DXL (μM)** | **Effect** | **CI** |
| --- | --- | --- | --- | --- |
| **MDA-MB-231** | 10.0 | 4.0 | 0.51 | 0.90350 |
|  | 20.0 | 4.0 | 0.65 | 0.53829 |
|  | 40.0 | 4.0 | 0.70 | 0.54999 |
| **4T1** | 10.0 | 4.0 | 0.50 | 0.94425 |
|  | 20.0 | 4.0 | 0.63 | 0.57227 |
|  | 40.0 | 4.0 | 0.71 | 0.48514 |


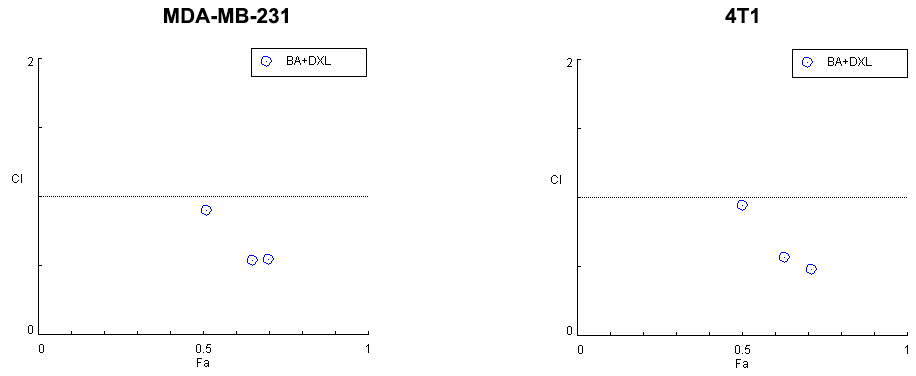


**Figure S1**. Combination index of BA and DXL in breast cancer cells.


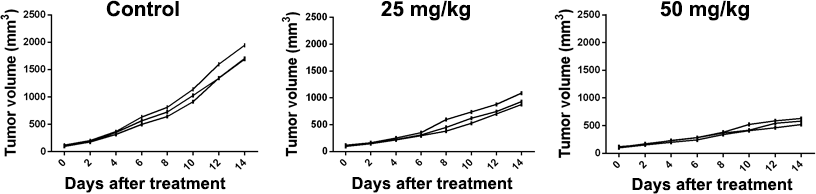


**Figure S2**. Individual data of tumor volumes in different treated group. 1×10^6^ 4T1 cells which was suspended in 100 μL of serum-free cell culture medium were injected into right flank of female Balb/c mouse to establish tumor mouse model. When the tumor volume reached about 100 mm^3^, the tumor-bearing mice were intraperitoneally administrated with vehicle, 25 mg/kg, or 50 mg/kg of BA every 2 days.
